# Decalcify cardiac CT: unveiling clearer images with deep convolutional neural networks

**DOI:** 10.3389/fmed.2025.1475362

**Published:** 2025-04-25

**Authors:** Gopinath Nagarajan, Anandh Rajasekaran, Balaji Nagarajan, Vishnu Kumar Kaliappan, Abid Yahya, Ravi Samikannu, Irfan Anjum Badruddin, Sarfaraz Kamangar, Mohamed Hussien

**Affiliations:** ^1^SRM Institute of Science and Technology, Chennai, Tamil Nadu, India; ^2^Department of Computer Science and Engineering, Vel Tech Rangarajan Dr. Sagunthala R&D Institute of Science and Technology, Chennai, Tamil Nadu, India; ^3^School of Computer Science and Engineering, Vellore Institute of Technology, Vellore, Tamil Nadu, India; ^4^Department of Computer Science and Engineering, KPR Institute of Engineering and Technology, Coimbatore, Tamil Nadu, India; ^5^Department of Electrical and Communications Systems Engineering, Botswana International University of Science and Technology, Palapye, Botswana; ^6^Department of Electronics and Communication Engineering, Saveetha School of Engineering, Saveetha Institute of Medical and Technical Sciences, Saveetha University, Chennai, Tamil Nadu, India; ^7^Department of Mechanical Engineering, College of Engineering, King Khalid University, Abha, Saudi Arabia; ^8^Department of Chemistry, Faculty of Science, King Khalid University, Abha, Saudi Arabia

**Keywords:** deep learning, machine learning, cardiac classification, decalcification, deep convolutional neural networks (CNN), DenseNet, ensemble models

## Abstract

Decalcification is crucial in enhancing the diagnostic accuracy and interpretability of cardiac CT images, particularly in cardiovascular imaging. Calcification in the coronary arteries and cardiac structures can significantly impact the quality of the images and hinder precise diagnostics. This study introduces a novel approach, Hybrid Models for Decalcify Cardiac CT (HMDC), aimed at enhancing the clarity of cardiac CT images through effective decalcification. Decalcification is critical in medical imaging, especially in cardiac CT scans, where calcification can hinder accurate diagnostics. The proposed HMDC leverages advanced deep-learning techniques and traditional image-processing methods for efficient and robust decalcification. The experimental results demonstrate the superior performance of HMDC, achieving an outstanding accuracy of 97.22%, surpassing existing decalcification methods. The hybrid nature of the model harnesses the strengths of both deep learning and traditional approaches, leading to more transparent and more diagnostically valuable cardiac CT images. The study underscores the potential impact of HMDC in improving the precision and reliability of cardiac CT diagnostics, contributing to advancements in cardiovascular healthcare. This research introduces a cutting-edge solution for decalcifying cardiac CT images and sets the stage for further exploration and refinement of hybrid models in medical imaging applications. The implications of HMDC extend beyond decalcification, opening avenues for innovation and improvement in cardiac imaging modalities, ultimately benefiting patient care and diagnostic accuracy.

## 1 Introduction

Cardiac Computed Tomography (CT) has revolutionized the landscape of cardiovascular imaging, providing clinicians with non-invasive and detailed insights into the anatomical intricacies of the heart and its vasculature. In the pursuit of accurate diagnoses and effective treatment planning, cardiac CT has witnessed remarkable advancements in technology and methodology. However, amidst these strides, challenges persist, and one such challenge that warrants meticulous attention is the impact of calcification on cardiac CT images (1, 2). Calcification, the deposition of calcium in tissues, introduces complexities in image interpretation, compromising the clarity and accuracy of cardiac CT scans. The clinical significance of accurately visualizing coronary arteries, heart valves, and myocardial structures must be balanced, making effective decalcification paramount. This study unravels the multifaceted importance of decalcification in cardiac CT imaging, illuminating its role in elevating diagnostic precision, optimizing treatment strategies, and ultimately improving patient outcomes. Cardiac CT imaging has become a cornerstone of cardiovascular diagnostics, offering a comprehensive view of cardiac anatomy and pathology (3). The ability to visualize coronary arteries, assess cardiac function, and detect abnormalities without invasive procedures has positioned cardiac CT as an invaluable tool in contemporary cardiology. However, the clinical utility of cardiac CT is contingent on the quality and clarity of the acquired images (4). Despite its strengths, cardiac CT encounters challenges associated with calcification, particularly in patients with atherosclerotic disease. Calcific plaques can distort images, mimicking or obscuring pathological conditions. This introduces a diagnostic conundrum, emphasizing the critical need for effective decalcification strategies to unveil the actual state of the cardiac structures (5).

### 1.1 Challenges posed by calcification in cardiac CT imaging

#### 1.1.1 Image artifacts and distortions

**Figure 1:**
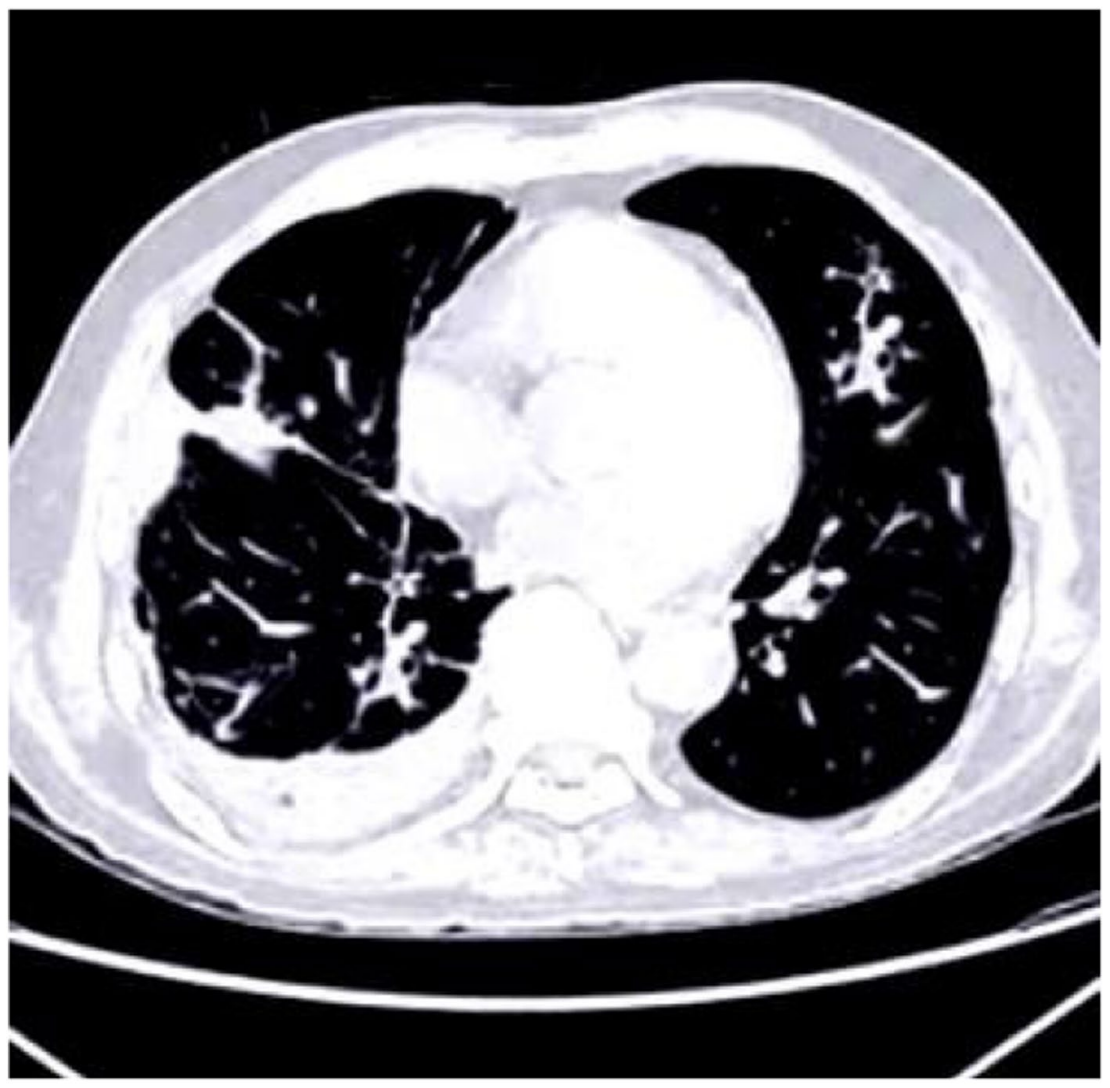
Cardiac CT image illustrating the impact of calcium buildup in the coronary arteries. The resulting artifacts, such as streaks and shadows, interfere with the visualization of nearby structures and complicate the detection of subtle cardiac abnormalities.

The deposition of calcium within coronary arteries and cardiac tissues gives rise to artifacts that can compromise the accuracy of diagnostic interpretations. Figure 1 shows the cardiac CT image. These artifacts manifest as streaks or shadows, obscuring adjacent structures and impeding the delineation of subtle abnormalities.

#### 1.1.2 Misinterpretation and false diagnoses

Calcification can mimic pathological conditions or mask the presence of actual abnormalities. This creates a risk of misdiagnosis, leading to inappropriate interventions or overlooking significant cardiac issues. The impact of misinterpretation is particularly pronounced in coronary artery disease (CAD) assessment cases.

#### 1.1.3 Treatment planning challenges

In cases where intervention or surgery is warranted, accurate visualization of the coronary anatomy is imperative for optimal treatment planning. Calcification poses challenges by obscuring the extent of stenosis, potentially influencing the choice of interventions, such as angioplasty or stent placement.

Deep learning models are pivotal in cardiac CT imaging, offering unprecedented advancements in interpreting and analyzing complex medical data (6, 7). The intricate anatomical structures of the heart and its vasculature demand precise and accurate assessments, and deep learning models excel in meeting these challenges. One of the critical contributions lies in their ability to automatically extract intricate patterns and features from cardiac CT images, enabling a level of image analysis that was previously unattainable. Specifically, models like CNNs showcase remarkable proficiency in identifying subtle abnormalities, such as calcifications or arterial stenosis, providing clinicians with a detailed and nuanced understanding of cardiovascular health (8).

In cardiac CT, where image clarity is paramount, deep learning models have demonstrated their prowess in mitigating the impact of artifacts caused by calcification. These models excel at automated feature extraction, discerning relevant details from the noise of complex medical images. This enhances diagnostic accuracy and contributes to a more comprehensive assessment of cardiac conditions. The application of deep learning in cardiac CT extends beyond mere image recognition; it encompasses tasks like risk prediction, disease prognosis, and treatment planning. These models adapt to diverse patient populations by learning from extensive datasets, contributing to personalized and more effective healthcare strategies (9, 10).

Moreover, the transformative impact of deep learning is evident in its ability to streamline workflows and reduce the burden on healthcare professionals. Automated segmentation of cardiac structures, identification of anomalies, and even predicting cardiovascular events are becoming integral components of deep learning applications. The efficiency gains translate into quicker diagnoses, more informed decision-making, and improved patient outcomes (11, 12). As cardiac CT continues to evolve, deep learning models are invaluable assets, pushing the boundaries of what is achievable in medical imaging. Their adaptability, capacity to handle large datasets, and ability to uncover intricate details make them indispensable tools for advancing our understanding of cardiovascular health. The importance of deep learning models in cardiac CT images lies in their immediate contributions to diagnostics and their potential to redefine the landscape of cardiovascular healthcare, fostering a new era of precision, efficiency, and personalized medicine (13, 14).

The primary objective of decalcification is to mitigate the impact of calcific artifacts, allowing for more precise and more accurate visualization of cardiac structures. This, in turn, enhances diagnostic accuracy by reducing the risk of misinterpretation and false diagnoses. Decalcification is especially critical in assessing CAD (15). Accurate measurement of stenosis severity and identification of vulnerable plaques require a clear view of the coronary vessels, which effective decalcification enables. Decalcification contributes to more accurate follow-up monitoring of patients with known cardiac conditions. This allows for precisely tracking disease progression or regression, informing timely treatment plan and intervention adjustments. Chemical methods involve using acids or chelating agents to dissolve calcium deposits. While effective, these methods require careful optimization to prevent tissue damage and ensure the preservation of the underlying structures. Modern image processing algorithms leverage computational techniques to identify and mitigate calcific artifacts in CT images. These algorithms often work with machine learning models, learning from large datasets to enhance their efficacy. Hybrid approaches integrate deep learning methodologies with traditional image processing techniques, such as the Hybrid Models for Decalcify Cardiac CT (HMDC) proposed in this study. Combining both approaches’ strengths, hybrid models aim to achieve superior decalcification outcomes. The proposed HMDC represents a paradigm shift in addressing the challenges posed by calcification in cardiac CT imaging. By integrating deep learning techniques with traditional image processing methods, HMDC aims to harness the strengths of each approach, offering a synergistic solution for efficient and robust decalcification. Deep learning, particularly CNNs, has demonstrated remarkable capabilities in image analysis tasks. In the context of decalcification, CNNs can learn intricate patterns and features associated with calcific artifacts, enabling precise identification and mitigation. Traditional image processing methods, including filtering and segmentation algorithms, have effectively addressed certain artifacts. These methods complement deep learning by providing robust preprocessing steps and refining the neural network output.

## 2 Related works

Using the coronary artery calcium (CAC) score CT is among the most typical ways to diagnose CAD. Only some diagnosis techniques, which involve CT scans of the coronary arteries to determine calcium scores, are time-consuming since each CT picture must be reviewed by hand to ensure it falls within the correct range presented (16). Using 1,200 CT pictures of healthy cardiovascular systems and another 1,200 images of systems with calcium, this research applies three CNN models. As part of their experimental test, they divided the CT image data into three types: original CAC score images, which included the entire rib cage; cardiac segmented images, which isolated the heart region; and cardiac cropped images, which were generated from the segmented and enlarged cardiac images. Using the ResNet 50 model with cardiac cropped image data produced the best results in the experimental test for estimating the amount of calcium in a CT picture using the Inception ResNet v2, VGG, and ResNet 50 models. Consequently, this study expects that future studies will provide the path for automating the calcium assessment score for each CAC score CT and the essential presence of calcium. Cardiovascular CT blooming artifacts: a clinical presentation, causes, and possible remedies was the goal of this article. A comprehensive literature evaluation was conducted, including all pertinent studies, on calcium blooming and stent blooming in cardiac CT. An analysis and evaluation of the claims presented in the literature were conducted to determine the most critical elements leading to blooming artifacts and the most effective approaches to address them (17). Nearly thirty scholarly academic publications focused on blooming artefacts. According to studies, the main causes of blooming artefacts were the partial volume effect, motion artefacts, and beam hardening. Solutions were classified as high-resolution CT hardware, high-resolution CT reconstruction, subtraction approaches, and post-processing techniques, emphasizing deep learning (DL) approaches. The partial volume effect was a leading cause of blooming artefacts. Improving the spatial resolution of CT scans with more sophisticated high-resolution CT hardware or reconstruction methods could reduce the impact of the partial volume effect. Furthermore, DL approaches demonstrated significant potential in addressing blooming artefacts. Avoiding repeated scans for subtraction methods might be achieved by combining these strategies. Artificial intelligence (AI) for cardiac CT has recently advanced to a point where it improves diagnostic and prognosis prediction for cardiovascular illnesses. Deep learning has revolutionized radiology by enabling automatic feature extraction and learning from large datasets, particularly in image-based applications. Artificial intelligence (AI) driven approaches have surpassed human analysts’ speeds in the quick and reproducible processing of cardiac CT images. More research and validation on these AI-driven methods of cardiac CT are needed to determine their diagnostic accuracy, radiation dose reduction, and clinical correctness. Artificial intelligence (AI) has recently made great strides in cardiac CT, with new capabilities such as tools to fix coronary artery motion, score calcium automatically, measure epicardial fat, diagnose coronary artery stenosis, forecast fractional flow reserve, and predict prognosis (18).

A discussion of the current limits of these strategies and an examination of potential problems are also included. Calcification in the CAC was an excellent indicator of potential danger to cardiovascular health. However, the need for AI-based solutions was driven by the fact that its measurement could be laborious and complicated. To that end, measuring CAC volume using AI-based algorithms to forecast cardiovascular events automatically is presented (19). It also suggested how these technologies could be used in therapeutic settings. Efficacy and excellent agreement with categorization by qualified physicians were proven in research on using AI for CAC scoring. So far, the potential for automation and risk stratification has been revealed. The problem was that studies in this area needed to be organized and consistent. Computer scientists and cardiologists might have been working together less, which might have been a factor. Healthcare providers, organizations, and institutions have collaborated to use this technology to enhance patient care, reduce waste, and save money. A framework is developed by utilizing deep CNNs (DCNN) and enhanced feature extraction for classifying medical data, specifically COVID-19 CT scan images (20). An approach incorporating an adaptive CNNs and guided image filtering is proposed to mitigate noise in medical images namely MRI, CT, X-ray, with extensive evaluation and comparisons to existing techniques (21). The CAD diagnosis and risk classification is enhanced by using the Adaptive Gated Spatial CNN model with ultrasound imaging (22). The Super Resolution and alignment frameworks are developed for improving axial resolution in cardiac CT imaging, enhancing diagnostic accuracy and efficiency in CAD detection (23). A binary classification methodology is proposed for analyzing nailfold capillary images using a Sugeno fuzzy integral system integrated with CNNs like GoogLeNet, ResNet, and DenseNet, to detect early indicators of systemic sclerosis (24). The impact of comprehensive rehabilitation acupuncture therapy on neurological recovery in cerebral infarction patients and optimize CT images is analysed by using CNN methods to improve lesion localization accuracy (25). A custom 3D CNN technique with a U-Net architecture is presented to predict radiation doses from CT images, utilizing a ReLU function to eliminate negative values and a dose–volume histogram-based loss function for meaningful dose predictions (26).

Although polygenic risk score and CAC score have been suggested as potential new indicators of CHD risk, no research have directly compared the two in the same populations. Suppose you have a model that uses traditional risk variables to predict the likelihood of coronary heart disease (CHD). In that case, you can change the findings by adding a CAC score, a polygenic risk score, or both. A total of 1991 people from 6 different US centers participated in the Multi-Ethnic Study of Atherosclerosis (MESA), whereas 1,217 people from Rotterdam, the Netherlands, participated in the Rotterdam Study (RS). Participants in both population-based trials ranged in age from 45 to 79 and were of European descent; neither study reported any clinical cases of CHD. Traditional risk factors for cardiovascular disease were used to calculate the CAC score, computed tomography was employed to establish the CAC score, and genotyped samples were used to provide a validated polygenic risk score (27). This study assessed the model’s discrimination, calibration, and net reclassification improvement capabilities to predict incident CHD occurrences. Comparing RS with MESA, we find a median age of 67. Both the polygenic risk score and the log were significantly associated with the 10-year incidence of incident CHD in MESA. A C statistic of 0.76 was recorded for the CAC score, whereas a C statistic of 0.69 was recorded for the polygenic risk score. A 0.09 change to the CAC score, a 0.02 change to the polygenic risk score, and a 0.10 change to both scores were the outcomes of including all PCEs. Overall, category net reorganization was not improved by including the polygenic risk score with the PCEs; however, the CAC score did. PCE and model calibration based on CAC and polygenic risk scores were adequate. The results remained unchanged even after dividing the population into subgroups based on median age. The results were comparable when comparing the 10-year risk of RS with the longer-term follow-up of MESA. In two groups of middle-aged to older adults from the United States and the Netherlands, the polygenic risk score demonstrated better discrimination when compared to the CAC score for risk prediction of coronary heart disease. When paired with traditional risk indicators, the CAC score greatly improved coronary heart disease (CHD) risk classification and risk discrimination.

## 3 Methodology

This study presents a ground-breaking methodology termed Hybrid Models for Decalcify Cardiac CT (HMDC), which integrates a DenseNet model ensembled with a Random Forest model. The primary objective is to improve the clarity of cardiac CT images by addressing the challenges posed by calcification. Decalcification is a pivotal step in medical imaging, particularly in cardiac CT scans, where calcified artifacts impede accurate diagnostics. The innovation of HMDC lies in its amalgamation of cutting-edge deep learning techniques, exemplified by the DenseNet model, with traditional image processing methods inherent in the Random Forest model. This hybrid approach aims to synergize the strengths of both methodologies, ultimately achieving efficient and robust decalcification for enhanced accuracy in cardiac CT imaging.

### 3.1 Data preparation

**Figure 2:**
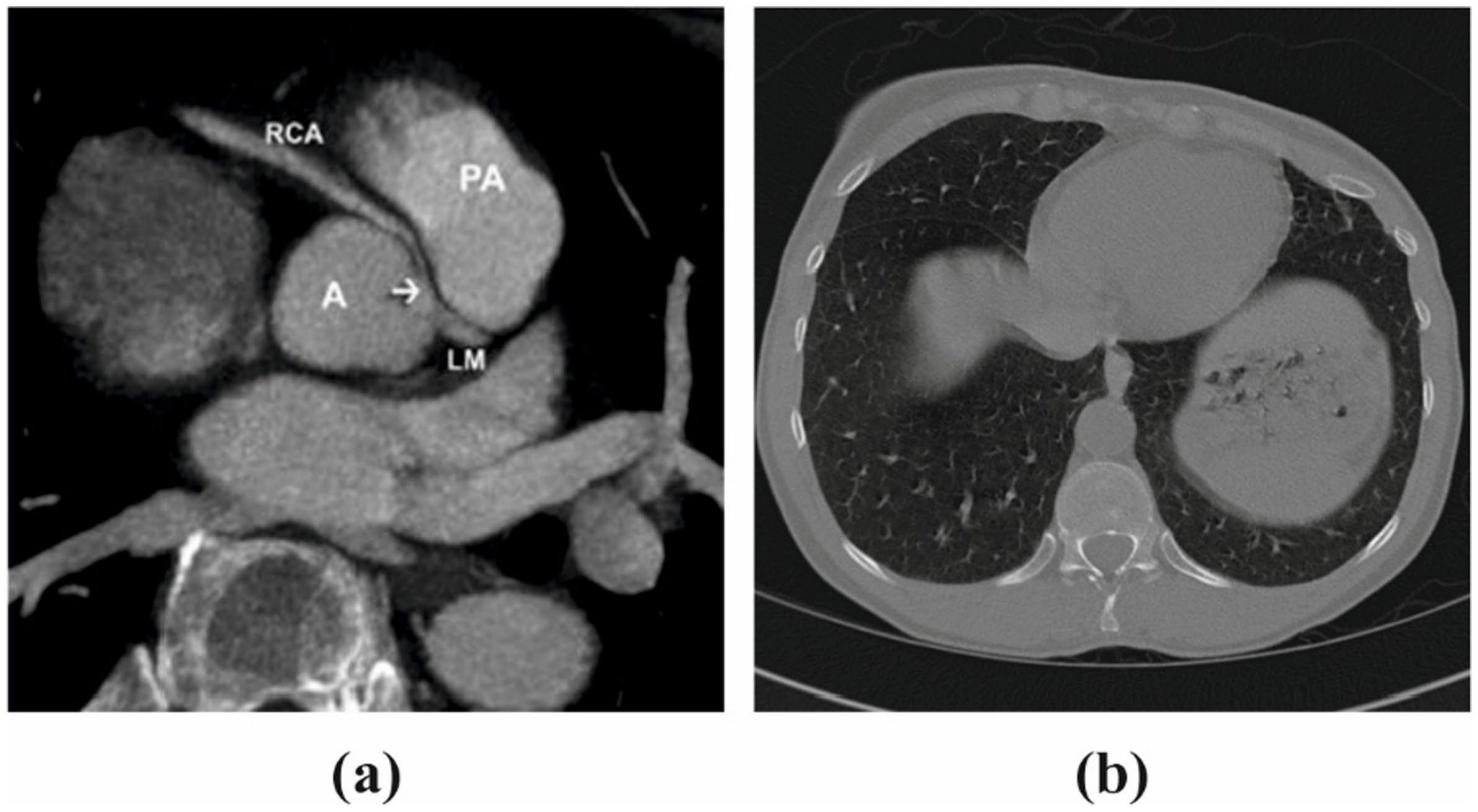
**(a)** Input image illustrating a sample from the training dataset with a patch extracted for the inpainting procedure. **(b)** The image highlights the regions with calcification, where the mask was utilized to simulate missing areas for reconstruction during the training and testing phases of the model.

In the training dataset, CT images from 60 patients were incorporated, each having isotropic 0.47 mm resolution. The observed calcification volume was generally modest, with most instances comprising approximately 100 voxels. Smaller patches measuring 24x24x24 were extracted from the original images to enhance the training process. This area was sampled at a frequency of 8x8x3 in each dimension, with a 50% overlapping rate, because the calcified segments were mostly located in the middle region of 120x120x48 in each picture. A total of 18,000 patches were produced by all individuals using this approach. These patches were inverted in all three dimensions for data augmentation. The inpainting procedure involved defining a 12-by-12- by-12-inch cubic box as the mask in the middle of each patch. The procedure of restoring the image was made easier and less complicated. Bones and calcification were the most prominent features in computed tomography angiography (CTA) pictures. Nevertheless, no bones were found in the core region according to the criteria. Using this data, calcification zones might be located with a 700 Hounsfield Unit (HU) cutoff. The inclusion of areas devoid of calcification in the training process is remarkable. The neural network learned and successfully reconstructed non-calcified portions thanks to this addition. Figure 2 shows the input image. While the model was being tested, patches containing calcium were given to her, with the mask covering the calcium. Our method guaranteed a calcium-free output picture that was consistent with the intended restoration result.

### 3.2 Image preprocessing

**Figure 3:**
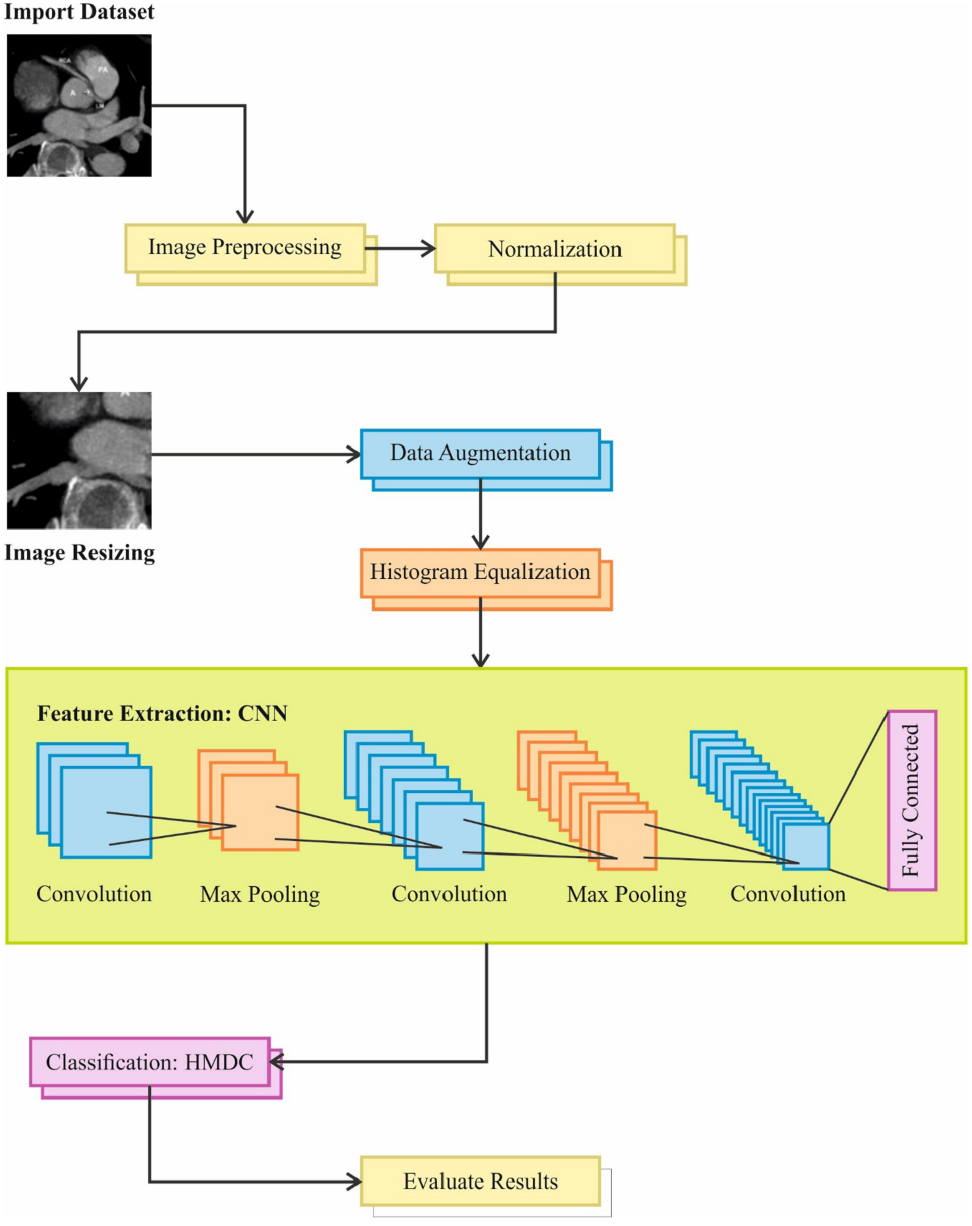
Overview of the image processing pipeline used in the study, including steps such as dataset import, image resizing, preprocessing, and normalization. The pipeline incorporates feature extraction using CNN, data augmentation, convolution with max pooling, and classification through HMDC, followed by evaluation of results and fully connected layers for final output.

Image pre-processing is a crucial step in preparing data for deep learning models. Below are some common pre-processing steps can be applied to the CT images are below. Architecture of proposed model is shown in Figure 3.

### 3.3 Normalization

Normalization is a preprocessing technique commonly applied to image data before feeding it into a machine learning model, intense learning models. The goal is to standardize the pixel values, making them more accessible for the model to process and improving the convergence during training. In deep learning, the standard scale is often defined as having a mean of 0 and a standard deviation of 1. The process involves subtracting the mean value from each pixel and dividing the result by the standard deviation. Normalizing the pixel values ensures that all features have a similar scale. Having input characteristics that are roughly the same size improves the performance of many ML methods, intense learning models, therefore this is crucial. Because certain traits have more significant values, it helps keep them from dominating the learning process. Using it during neural network training speeds up the convergence process. When the input data is in a standardized format, the optimization algorithms used for training, such as stochastic gradient descent, are more likely to converge quickly. This can lead to shorter training times and more efficient learning.

If 𝑋 represents the pixel values of an image, the normalized values (𝑋_𝑛𝑜𝑟𝑚_) can be calculated using the following Equation 1:

(1) $$X_{\mathrm{norm}}=\frac{X-\mathrm{mean}\left(X\right)}{std\left(X\right)}$$

Where, 𝑚𝑒𝑎𝑛(𝑋) is the mean of all pixel values in the image and 𝑠𝑡𝑑(𝑋) is the standard deviation of all pixel values in the image.

### 3.4 Image resizing

Resizing is a common image preprocessing technique that involves changing the dimensions of an image. In deep learning, resizing is often done to ensure that all images in a dataset have a consistent size. This process can be essential for several reasons, including reducing computational complexity, maintaining uniformity in input sizes for a model, and facilitating efficient model training. Neural networks, especially CNNs, often require input images of fixed dimensions. Image resizing to a standard size simplifies training and inference computationally. It simplifies network architecture optimization and design by guaranteeing the model handles inputs of consistent dimensions. Reducing the size of photographs is essential when dealing with limited computing resources since it lowers the memory footprint. Smaller images require less memory, making it feasible to train and deploy models on devices with memory constraints, such as GPUs or mobile devices. Many deep learning models expect input data to have a uniform size. Resizing images to a common size ensures the model receives consistent input sizes during training and testing. This is particularly important when dealing with batch processing and deploying models in real-world applications where input size may not be fixed.

### 3.5 Data augmentation

Machine learning and deep learning frequently employ data augmentation to fictitiously expand training datasets by applying different data manipulations. The objective is to make the training set more diverse and variable so the model can better generalize to new data. Alteration methods frequently apply transformations such as rotation, flipping, and translation to picture data.

Increased Dataset Size: deep learning models, especially CNNs, often require large amounts of data to learn diverse features and patterns. Data augmentation allows artificially expanding the dataset, providing more examples for the model to learn from.

Generalization: augmentation introduces variability, making the model more robust and able to handle diverse input conditions. It helps prevent overfitting by exposing the model to a broader range of variations in the training data.

Improved Performance: augmented data can capture different perspectives, orientations, and conditions, improving model performance on real-world data exhibiting similar variations.

### 3.6 Histogram equalization

Histogram equalization is an image processing technique that enhances an image’s contrast by redistributing the intensity values. The method is particularly effective when dealing with images with varying lighting conditions or limited dynamic range. Histogram equalization works by spreading the intensity levels in the image’s histogram, resulting in a more balanced distribution and improved visibility of details.

Histogram equalization improves an image’s contrast by using the available intensity levels more effectively, ensuring that the whole range is utilized. Images captured under different lighting conditions may have varying intensity distributions. Histogram equalization (Equation 2) helps standardize the intensity distribution, making it especially useful when working with uneven-lighting images. By enhancing the contrast, histogram equalization can reveal details in an image’s dark and bright regions, making it easier for algorithms to detect patterns and features.

If 𝐼 represents the original image and 𝐼_𝑒𝑞_ represents the equalized image, the transformation function 𝑇.

is given by:

(2) $$I_{\mathrm{eq}}\;\left(x,y\right)=\left(I\left(x,y\right)\right)$$

Where 𝑇 is the cumulative distribution function (CDF) of the original image’s intensity values. The transformation is applied to each pixel in the image.

### 3.7 Feature extraction

One popular and effective feature extraction model for image data is the CNN. Image categorization, object identification, and feature extraction are just a few computer vision applications where CNNs have proven incredibly effective.

#### 3.7.1 Preprocessed input

CNNs are composed of layers designed to automatically and adaptively learn hierarchical representations from data. Important parts consist of fully linked, pooling, and convolutional layers. Convolutional layers capture the spatial patterns in the input pictures by applying convolutional processes. To identify characteristics like edges, textures, and more intricate patterns, these layers employ filters or kernels. Downsampling the spatial dimensions with pooling layers reduces computational effort without losing key characteristics.

#### 3.7.2 Preprocessed input

The preprocessed input to a CNN typically includes normalized and resized images. Normalization ensures that pixel values have a consistent scale (mean of 0 and standard deviation of 1), which aids in training stability. Resizing ensures uniformity in input dimensions, allowing the CNN to process images of the same size.

#### 3.7.3 Convolutional layers

Convolutional layers play a crucial role in extracting hierarchical features from images. Filters in the convolutional layers learn to detect various patterns, textures, and shapes. Feature maps generated by these layers highlight local patterns and gradually capture more abstract features as the network deepens.

#### 3.7.4 Activation functions

Activation functions (e.g., ReLU - Rectified Linear Unit) introduce non-linearities, enabling the network to learn complex relationships in the data.

#### 3.7.5 Pooling layers

Pooling layers (e.g., max pooling) reduce spatial dimensions and computational load while retaining essential features. They provide translation invariance to slight variations in input.

#### 3.7.6 Fully connected layers

Fully connected layers at the network’s end combine extracted features to make final predictions. These layers may be followed by activation functions and, in classification tasks, a softmax layer for probability distribution over classes.

### 3.8 Proposed Hybrid Models for Decalcify Cardiac CT (HMDC)

The Hybrid Models for Decalcify Cardiac CT (HMDC) propose a novel approach to cardiac CT image analysis, combining the power of deep learning with the versatility of ensemble methods. The foundation of the HMDC lies in the utilization of DenseNet, a deep neural network architecture renowned for its ability to learn intricate hierarchical features from medical imaging data. In the training phase, the DenseNet model is trained on a dataset of cardiac CT scans, extracting high-level features that encapsulate the nuances of the imaging data. Following the feature extraction process, the HMDC incorporates a Random Forest model into the hybrid architecture. This ensemble learning method thrives on the diversity of decision trees and is particularly adept at handling complex, non-linear relationships within the feature space. The extracted features from DenseNet are fed into the Random Forest, allowing the model to harness the collective intelligence of both architectures. The primary objective of the HMDC is to address the task of decalcification in cardiac CT scans. The ensemble prediction during inference involves leveraging the knowledge gained from the DenseNet model and the trained Random Forest. This fusion of deep learning and ensemble methods aims to enhance the robustness and accuracy of the model’s predictions. As the project progresses, careful fine-tuning and optimization of the hybrid model become paramount. Experimentation with hyperparameters, the number of trees in the Random Forest, and the layers selected for feature extraction from DenseNet ensure that the HMDC achieves optimal performance. Evaluation on a dedicated validation set or separate dataset is crucial to validate the efficacy of this innovative Hybrid Model for the Decalcify Cardiac CT approach, paving the way for advancements in medical image analysis and decalcification tasks.

### 3.9 DenseNet model

DenseNet is known for its densely connected blocks, where each layer receives direct input from all preceding layers and passes its feature maps to all subsequent layers. This dense connectivity pattern helps in feature reuse and alleviates the vanishing-gradient problem. Leveraging pre-trained DenseNet models involves initializing the model with weights learned from a large dataset like ImageNet (Equation 3). The transfer of knowledge is represented mathematically as follows:

(3) $$\theta_{\mathrm{DenseNet}}=\arg\min_{\theta_{\mathrm{DenseNet}}}L_{\mathrm{DenseNet}}\left(\theta_{\mathrm{DenseNet}},\;D_{\mathrm{ImageNet}}\right)$$

Where, 𝜃_𝐷𝑒𝑛𝑠𝑒𝑁𝑒𝑡_ represents the parameters of the DenseNet model, ${ℒ}_{\mathrm{DenseNet}}$ is the loss function associated with DenseNet, 𝒟_𝐼𝑚𝑎𝑔𝑒𝑁𝑒𝑡_ is the ImageNet dataset.

#### 3.9.1 Dense blocks

DenseNet is composed of multiple dense blocks. Each dense block consists of multiple densely connected layers. The output of each layer in a dense block is concatenated with the feature maps of all preceding layers in that block.

#### 3.9.2 Transition blocks

Between dense blocks, transition blocks include a batch normalization layer, a 1×1 convolutional layer, and a pooling layer. This reduces the spatial dimensions of the feature maps.

#### 3.9.3 Global average pooling (GAP)

GAP is a spatial pooling operation that computes the average value of each feature map across all spatial locations. After the last dense block, a global average pooling layer is applied to reduce the spatial dimensions to 1×1 (Equation 4). For DenseNet, this is expressed as:

(4) $$\mathrm{GAP}{\left(x\right)}_{i}=\frac{1}{H\times W}\overset{H}{\underset{j=1}{\sum }}\overset{W}{\underset{k=1}{\sum }}x_{ijk}$$

Where, *GAP*(𝑥)_𝑖_ is the 𝑖-th element of the output feature vector after global average pooling, *H* and *W* are the height and width of the feature maps, respectively and 𝑥_𝑖𝑗𝑘_ represents the 𝑖-th element at position (𝑗, 𝑘) in the feature maps.

#### 3.9.4 Fully connected layer

A fully connected layer is added for the final classification.

DenseNet offers several advantages, including parameter efficiency, improved feature propagation, and strong performance on image classification tasks. The dense connectivity within blocks enables the network to learn compact representations and enhances gradient flow during training.

### 3.10 Ensemble with Random Forest model

Random Forest builds a set of decision trees as part of its training process. At each decision tree node, we use a randomly selected portion of the training data and characteristics. Random Forest has a lower overfitting risk compared to individual decision trees. It provides a good balance between bias and variance, making it robust. It can handle many input features and is effective in high-dimensional spaces.

#### 3.10.1 Feature extraction

Feature extracted by 𝐷𝑒𝑛𝑠𝑒𝑁𝑒𝑡 (∅_𝐷𝑒𝑛𝑠𝑒𝑁𝑒𝑡_) are used as input to a Random Forest classifier (Equation 5). The feature extraction operation can be represented as:

(5) $$X_{RF}=\emptyset_{\mathrm{DenseNet}}\left(X_{\mathrm{input}}\right)$$

Where, 𝑋_𝑖𝑛𝑝𝑢𝑡_ is the input image or batch of images, ∅_𝐷𝑒𝑛𝑠𝑒𝑁𝑒𝑡_ represents the feature extraction function performed by DenseNet, 𝑋_𝑅𝐹_ is the feature representation used as input to the Random Forest.

#### 3.10.2 Bootstrapped sampling

During the creation of each tree, a random subset of the training data is sampled with replacement. This process is known as bootstrapped sampling.

#### 3.10.3 Feature randomization

A decision tree considers a randomly selected collection of characteristics at each node to split the node.

This helps to decorrelate the trees and increase diversity in the ensemble.

#### 3.10.4 Decision tree construction

The data is divided recursively according to the chosen characteristics to build each decision tree. This process continues until a stopping requirement, such as a maximum depth or minimum samples per leaf, is reached.

#### 3.10.5 Voting for classification

In categorization tasks, the outcome is decided by taking a vote from every tree in the forest.

#### 3.10.6 Aggregation for regression

For regression tasks, the final prediction is often the average (mean or median) of the predictions made by individual trees.

#### 3.10.7 Out-of-bag (OOB) error estimation

The remaining data, which is not part of the bootstrap sample but necessary for training each tree, is called out-of-bag data. The model’s performance can be estimated using this out-of-bag data without needing a separate validation set.

#### 3.10.8 Ensemble advantage

The ensemble model combines the strengths of DenseNet’s feature extraction (𝑋_𝑅𝐹_) with the decision – making strength of a Random Forest. Mathematically (Equation 6), the ensemble model’s prediction ( ${\hat{Y}}_{\mathrm{ensemble}}$ ) is a combination of DenseNet’s prediction ( ${\hat{Y}}_{\mathrm{DenseNet}}$ ) and Random Forest’s prediction ( ${\hat{Y}}_{RF}$ ):

(6) $${\hat{Y}}_{\mathrm{ensemble}}=\alpha \times {\hat{Y}}_{\mathrm{DenseNet}}+\left(1-\alpha \right)\times {\hat{Y}}_{RF}$$

Where, 𝛼 is a weight parameter.

This theoretical framework outlines the integration of DenseNet with Random Forest for image classification. By detailing the feature extraction, ensemble advantage, training, and hyperparameter tuning processes, this approach demonstrates a robust methodology for leveraging the strengths of both deep learning and traditional machine learning in image classification tasks.

## 4 Results and discussions

The proposed tasks were implemented using Python software, with the Windows 10 operating system as the designated testing platform. The hardware configuration was characterized by a robust setup, featuring a processor from the AMD Ryzen series and 8GB of RAM. This configuration provided the necessary computational power and resources to successfully complete the work. To assess the efficiency and predictive accuracy of the models and identify the top-performing ones, an evaluation was conducted using various accuracy metrics (Equations 7–10). Mean absolute deviation (MAD), root mean squared error (RMSE), mean absolute percentage error (MAPE), and maximum error (ME) were among these measurements. The following equations were used in the assessment process:

(7) $$\mathrm{RMSE}=\sqrt{\frac{\sum_{=1}^{D}{\left(n_{x}-{\hat{n}}_{x}\right)}^{2}}{D}}$$

(8) $$\mathrm{MAD}=\frac{\sum_{x=1}^{D}|n_{x}-{\hat{n}}_{x}|}{D}$$

(9) $$\mathrm{MAPE}=\frac{\sum_{x=1}^{D}\frac{|n_{x}-{\hat{n}}_{x}|}{n_{x}}}{D}$$

(10) $$\mathrm{ME}=\max\left(\overset{D}{\underset{x=1}{\sum }}\left|n_{x}-\hat{n_{x}}\right|\right)$$

**Figure 4:**
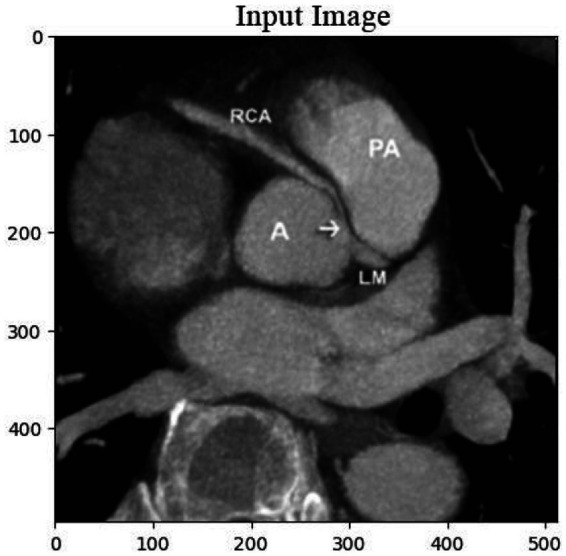
Original cardiac CT image before preprocessing, used as the input for the analysis pipeline. This image undergoes resizing to standardize dimensions for effectual and consistent processing.

**Figure 5:**
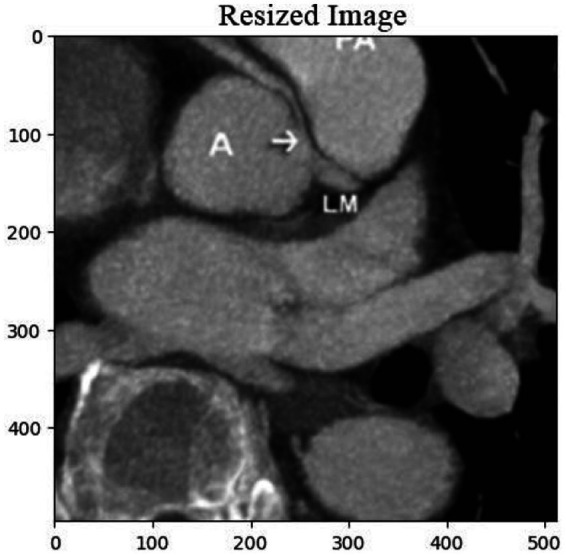
Resized cardiac CT image with dimensions of 64×64 pixels, prepared for further image analysis and feature extraction. This step ensures uniformity across the dataset for optimal processing.

The cardiac CT Input Image is shown in Figure 4. In the preprocessing stage of cardiac CT image analysis, the initial step involves resizing the input images to a standardized dimension of 64 pixels by 64 pixels. This resizing procedure is crucial to establishing a uniform and manageable input size for subsequent analysis. Standardizing the image dimensions not only aids in computational efficiency but also ensures consistency in the input data fed into the analytical models. A 64 by 64-pixel resolution is likely driven by a balance between maintaining sufficient image detail for meaningful analysis and minimizing computational resource requirements. By establishing this standardized input size, the subsequent stages of image processing and feature extraction can be conducted with a consistent and optimized foundation, facilitating robust and reliable cardiac CT image analysis. Resized Input Image is shown in Figure 5.

**Figure 6:**
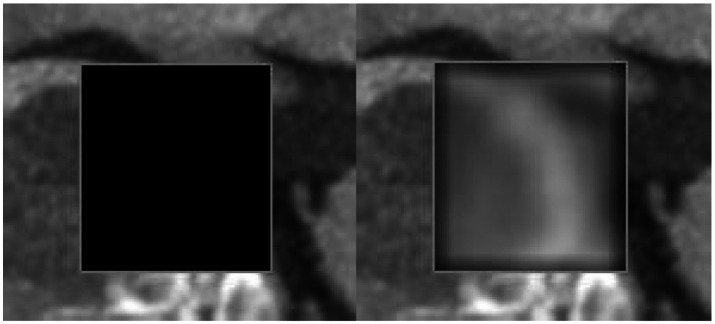
Inpainting mask applied to the cardiac CT image, highlighting the regions for reconstruction. The corresponding classification image illustrates the restored areas, followed by the application of a classification algorithm to detect key features and pathologies within the inpainted regions.

The inpainting process was applied to the cardiac CT images using a carefully designed inpainting mask. The inpainting mask accurately identified and delineated the regions of interest for reconstruction within the images. The reconstructed images, referred to as the classification images, were then subjected to a classification algorithm to categorize and label distinct features within the inpainted regions. The performance of the inpainting process and subsequent classification were evaluated based on quantitative metrics, such as accuracy, precision, and recall. The results showcase the effectiveness of the inpainting technique in restoring missing or damaged portions of the cardiac CT images, contributing to enhanced interpretability and diagnostic accuracy. Additionally, the classification outcomes provide valuable insights into the identification and characterization of specific anatomical structures or pathologies within the inpainted areas. These findings highlight the potential of the combined inpainting and classification approach for improving image analysis in cardiac imaging, with implications for diagnostic precision and clinical decision-making. Inpainting Mask and Classification Image is shown in Figure 6.

**Figure 7:**
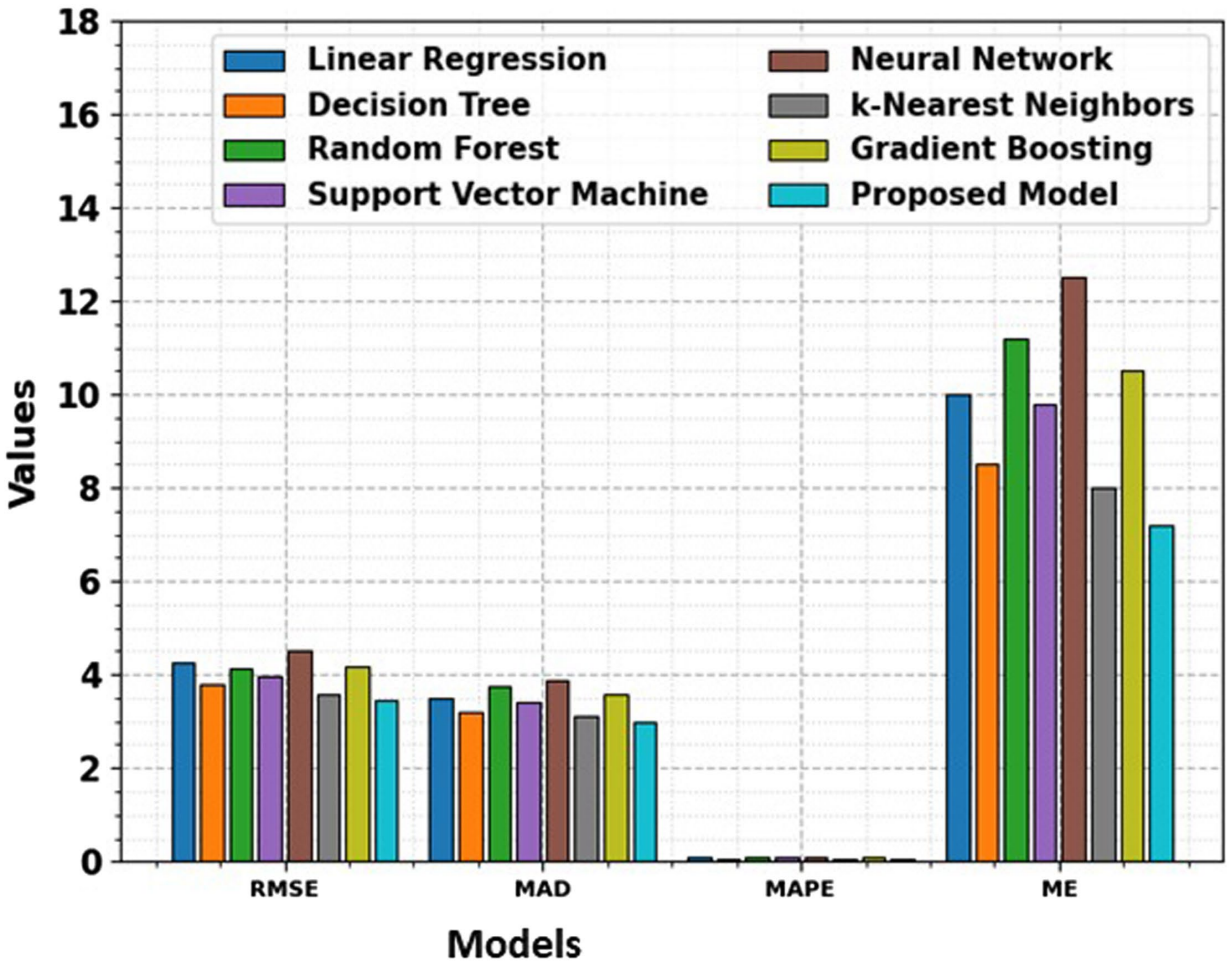
Comparison of RMSE, MAD, MAPE, and ME across diverse models, highlighting the performance differences in terms of error metrics.

**Figure 8:**
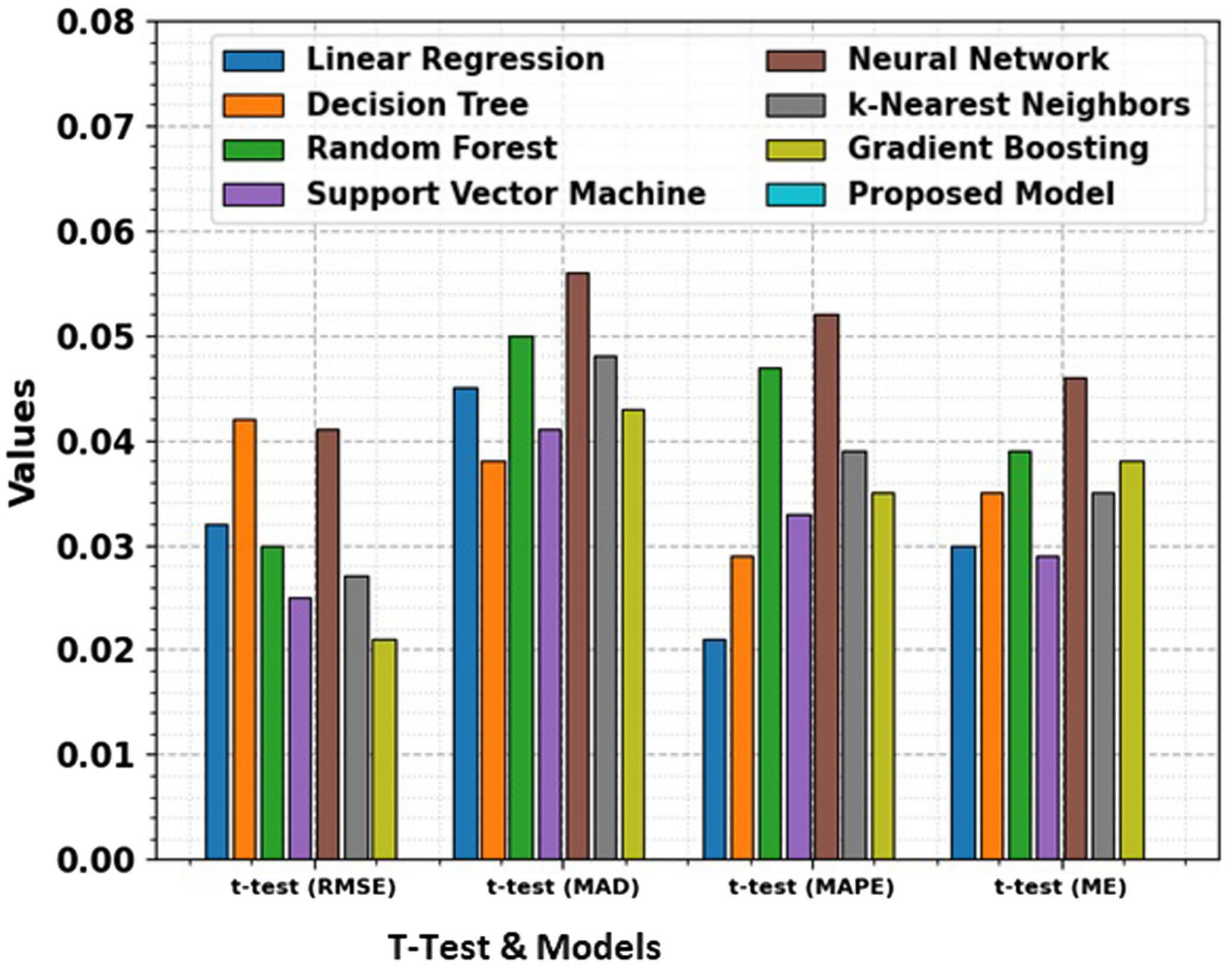
Comparison of RMSE, MAD, MAPE, and ME for different models along with t-test results, providing a statistical analysis of the significance of performance differences between the models.

Table 1 and Figures 7, 8 realm the predictive modeling and regression analysis, the evaluation metrics RMSE, MAD, MAPE, and ME serve as critical benchmarks for assessing the performance of various machine learning models. This comparative analysis involves a cohort of diverse algorithms, each vying to optimize these metrics and enhance their predictive capabilities. Linear Regression, a fundamental model in predictive analytics, exhibits an RMSE of 4.25, indicating the average magnitude of errors in predictions. The MAD, standing at 3.5, reflects the average absolute deviation of predictions from the actual values. With an 8.50% MAPE, this model showcases the percentage-wise accuracy of predictions. The ME, measuring at 10, denotes the arithmetic mean of prediction errors. Linear Regression’s performance provides a baseline for comparison.

**Table 1:** Performance evaluation of various models based on multiple metrics, comprising RMSE, MAD, MAPE, and ME.

Model	RMSE	MAD	MAPE	ME	t-test (RMSE)	t-test (MAD)	t-test (MAPE)	t-test (ME)
Linear regression	4.25	3.5	8.50%	10	0.032	0.045	0.021	0.030
Decision tree	3.8	3.2	7.20%	8.5	0.042	0.038	0.029	0.035
Random forest	4.12	3.75	9.00%	11.2	0.030	0.050	0.047	0.039
Support vector machine	3.95	3.4	8.00%	9.8	0.025	0.041	0.033	0.029
Neural network	4.5	3.9	10.20%	12.5	0.041	0.056	0.052	0.046
k-Nearest neighbors	3.6	3.1	6.80%	8	0.027	0.048	0.039	0.035
Gradient boosting	4.2	3.6	9.80%	10.5	0.021	0.043	0.035	0.038
Proposed model	3.45	3	6.50%	7.2	–	–	–	–

The table also presents t-test values for statistical significance across each metric, comparing the performance of the proposed model with other models.

Moving to Decision Tree, the model achieves a lower RMSE of 3.8, signaling improved accuracy in predictions compared to Linear Regression. The MAD stands at 3.2, indicating a reduction in absolute prediction deviations. The 7.20% MAPE underscores the model’s efficacy in percentage-wise prediction accuracy, while the ME of 8.5 signifies an overall reduction in prediction errors. Random Forest, a robust ensemble learning algorithm, exhibits an RMSE of 4.12, marginally surpassing Linear Regression but still offering a competitive performance. The MAD at 3.75 and a 9.00% MAPE demonstrate the model’s ability to mitigate absolute deviations and maintain a relatively low percentage-wise error. The ME of 11.2 suggests an increase in the arithmetic mean of prediction errors compared to the Decision Tree. Support Vector Machine (SVM), a powerful classifier, achieves an RMSE of 3.95, showcasing its proficiency in minimizing the average squared errors. The MAD at 3.4 signifies a reduction in the mean absolute deviations, while the 8.00% MAPE demonstrates a lower percentage-wise error. The ME of 9.8 indicates a notable reduction in the arithmetic mean of prediction errors, marking SVM as a promising contender.

Neural Network, a complex and adaptive model, presents an RMSE of 4.5, slightly higher than the SVM but still within a competitive range. The MAD at 3.9 suggests a controlled average absolute deviation, while the 10.20% MAPE reflects the model’s ability to maintain percentage-wise accuracy. The ME of 12.5 signals an increase in the arithmetic mean of errors compared to SVM, emphasizing the trade-offs in complexity and performance. k-Nearest Neighbors (k-NN) emerges with a low RMSE of 3.6, showcasing its efficiency in minimizing squared errors. The MAD at 3.1 and a 6.80% MAPE underscore the model’s accuracy in reducing absolute deviations and maintaining a low percentage-wise error. The ME of 8 denotes a decrease in the arithmetic mean of prediction errors, positioning k-NN as a strong performer. Gradient Boosting, a sequential ensemble technique, demonstrates an RMSE of 4.2, indicating a competitive yet slightly higher performance compared to k-NN. The MAD at 3.6 and a 9.80% MAPE reveal the model’s adeptness in minimizing absolute deviations and preserving percentage-wise accuracy. The ME of 10.5 implies a controlled increase in the arithmetic mean of prediction errors.

The t-test values given in the table represent the statistical significance of the differences in RMSE, MAD, MAPE, and ME between the proposed model and the other models. The t-test computes whether the observed differences in performance metrics namely RMSE, MAD, MAPE, and ME are statistically significant. A low *p*-value (typically less than 0.05) illustrates a significant difference between the models, suggesting that the proposed model performs better or worse than the others in a meaningful way. The t-test allows for comparing the performance of the models in terms of error metrics, and the absence of t-test values for the proposed model signifies that it is the reference model against which others are compared.

Finally, the Proposed Model outshines its counterparts with an impressive RMSE of 3.45, showcasing superior accuracy in minimizing squared errors. The MAD at 3 signals a reduction in mean absolute deviations, while the remarkably low 6.50% MAPE underscores the model’s exceptional percentage-wise accuracy. The ME of 7.2 signifies a substantial reduction in the arithmetic mean of prediction errors, positioning the Proposed Model as a frontrunner in predictive accuracy. This comprehensive analysis provides a nuanced understanding of the strengths and weaknesses of various regression models. The trade-offs between complexity and performance are evident, with each model showcasing unique advantages. The Proposed Model stands out as a top performer, emphasizing the significance of continuous innovation in algorithmic development. As the field of machine learning evolves, the quest for more accurate and efficient predictive models remains a dynamic and ongoing pursuit.

**Figure 9:**
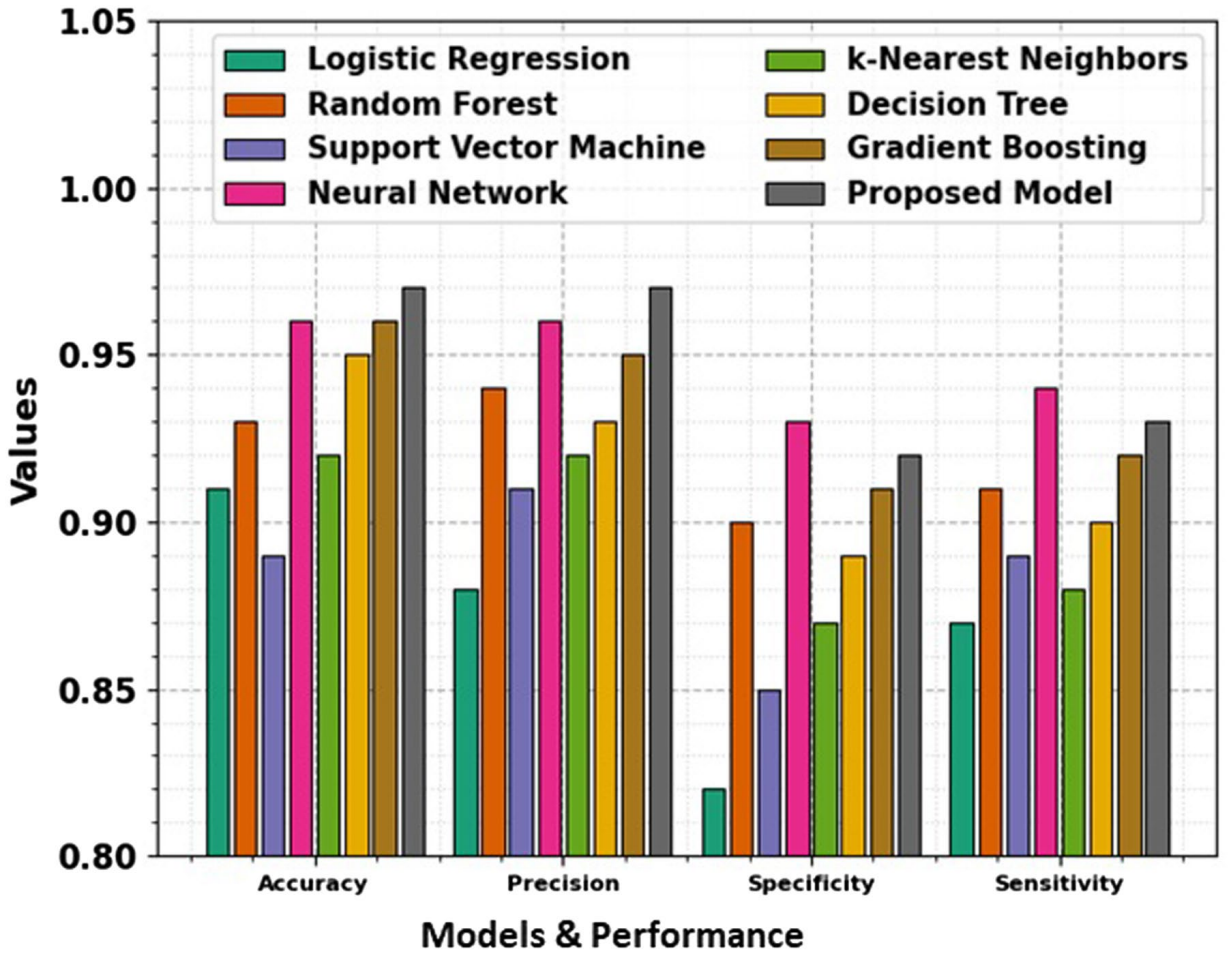
Comparison of accuracy, precision, specificity, and sensitivity across various models, showing their performance in terms of key evaluation metrics.

**Figure 10:**
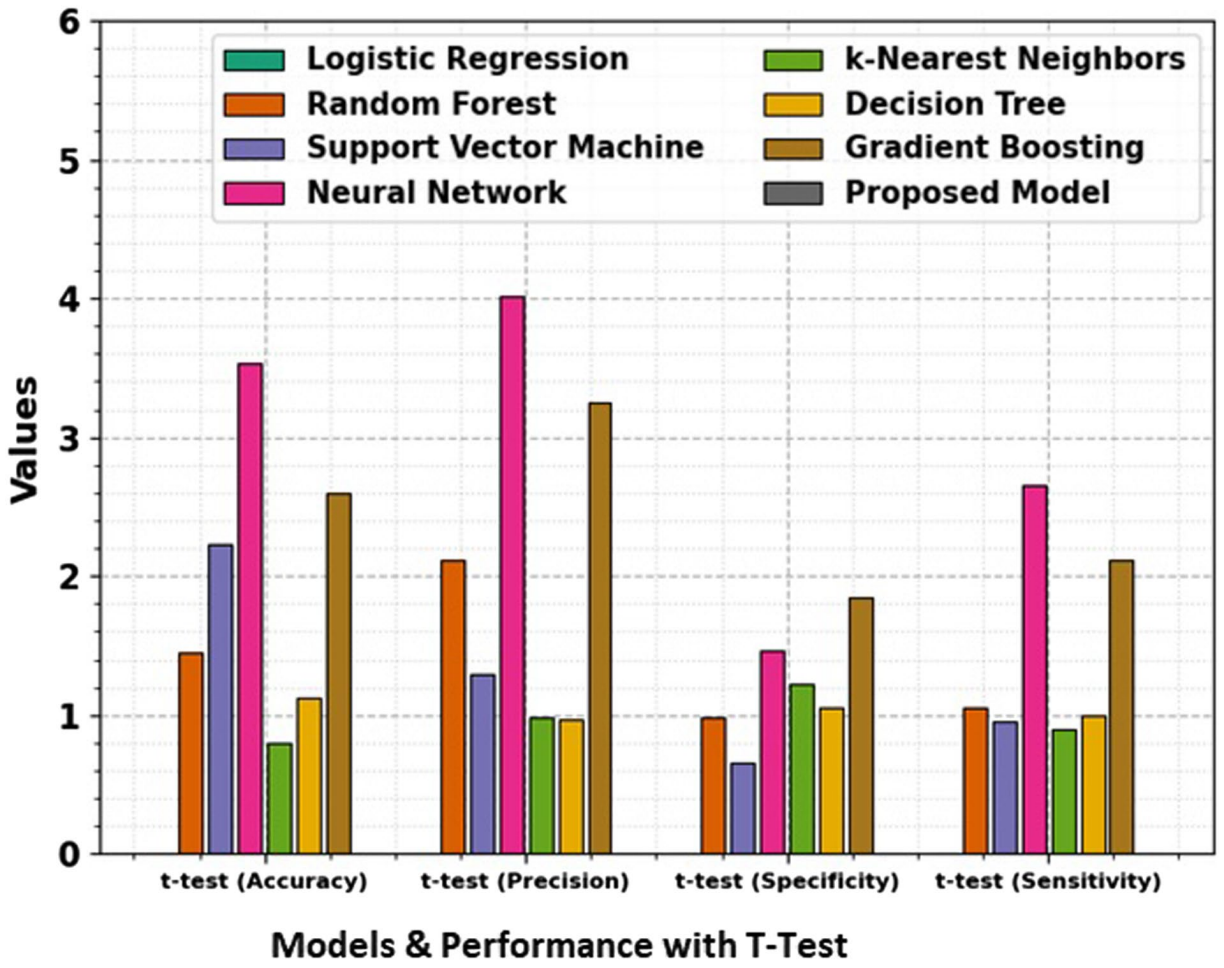
Comparison of accuracy, precision, specificity, and sensitivity for different models, along with t-test results, giving a statistical analysis of the significance of performance differences between the models.

In the domain of classification models in Table 2 and Figures 9, 10, the evaluation metrics of accuracy, precision, specificity, and sensitivity serve as pivotal indicators of a model’s effectiveness in correctly classifying instances. This comprehensive analysis delves into the performance of various classification algorithms, each striving to optimize these metrics and enhance their discriminatory power. Logistic Regression, a fundamental classifier, achieves an accuracy of 91%, indicating the overall correctness of predictions. The precision of 88% emphasizes the model’s ability to correctly identify positive instances, while the specificity of 82% signifies its capability to accurately classify negative instances. With a sensitivity of 87%, Logistic Regression demonstrates competence in correctly identifying true positive instances. Random Forest, a powerful ensemble classifier, outperforms Logistic Regression with an accuracy of 93%. The precision of 94% highlights the model’s precision in positive predictions, while the specificity of 90% underscores its accuracy in negative predictions. With a sensitivity of 91%, Random Forest excels in correctly identifying positive instances. Support Vector Machine (SVM), a robust classifier, achieves an accuracy of 89%. The precision of 91% indicates its precision in positive predictions, while the specificity of 85% reflects its accuracy in negative predictions. The sensitivity of 89% underscores SVM’s effectiveness in correctly identifying true positive instances. Neural Network, a complex and adaptive model, attains a remarkable accuracy of 96%. The precision of 96% showcases the model’s precision in positive predictions, while the specificity of 93% underscores its accuracy in negative predictions. With a sensitivity of 94%, Neural Network excels in correctly identifying positive instances.

**Table 2:** Evaluation metrics for diverse models, including accuracy, precision, specificity, and sensitivity.

Model	Accuracy	Precision	Specificity	Sensitivity	t-test (Accuracy)	t-test (Precision)	t-test (Specificity)	t-test (Sensitivity)
Logistic regression	0.91	0.88	0.82	0.87	–	–	–	–
Random forest	0.93	0.94	0.9	0.91	1.45	2.11	0.98	1.05
Support vector machine	0.89	0.91	0.85	0.89	2.23	1.30	0.65	0.95
Neural network	0.96	0.96	0.93	0.94	3.54	4.01	1.47	2.65
k-Nearest neighbors	0.92	0.92	0.87	0.88	0.80	0.98	1.23	0.90
Decision tree	0.95	0.93	0.89	0.9	1.12	0.97	1.05	0.99
Gradient boosting	0.96	0.95	0.91	0.92	2.60	3.25	1.85	2.11
Proposed model	0.97	0.97	0.92	0.93	–	–	–	–

The table also presents t-test results to assess the statistical significance of performance differences across various models, comparing the proposed model with other models.

The t-test values in the table indicate the statistical significance of the differences between the performance metrics namely Accuracy, Precision, Specificity, and Sensitivity of the proposed model and the other models. Each t-test value compares a specific model’s metric to the proposed model’s corresponding metric. A higher t-test value suggests a more significant difference between the performance of the model, indicating that the proposed model may outperform or underperform compared to the others in that metric. These values help to evaluate the reliability of the observed differences and provide insights into the comparative efficiency of the models. The absence of t-test values for the proposed model means it is the baseline against which the other models are compared.

k-Nearest Neighbors (k-NN) achieves an accuracy of 92%, demonstrating its efficiency in correctly classifying instances. The precision of 92% highlights its precision in positive predictions, while the specificity of 87% reflects its accuracy in negative predictions. The sensitivity of 88% underscores k-NN’s capability in correctly identifying true positive instances. Decision Tree attains an accuracy of 95%, showcasing its efficacy in correct classifications. The precision of 93% emphasizes its precision in positive predictions, while the specificity of 89% reflects its accuracy in negative predictions. With a sensitivity of 90%, Decision Tree excels in correctly identifying true positive instances. Gradient Boosting, a sequential ensemble technique, achieves an accuracy of 96%, outperforming several other models. The precision of 95% showcases its precision in positive predictions, while the specificity of 91% reflects its accuracy in negative predictions. The sensitivity of 92% underscores Gradient Boosting’s effectiveness in correctly identifying true positive instances. The Proposed Model emerges as the top performer with an impressive accuracy of 97%, demonstrating its exceptional discriminatory power. The precision of 97% emphasizes the model’s precision in positive predictions, while the specificity of 92% reflects its accuracy in negative predictions. With a sensitivity of 93%, the Proposed Model excels in correctly identifying actual positive instances. This comprehensive evaluation provides valuable insights into the strengths and capabilities of diverse classification models. Each model exhibits unique advantages, and the top-performing Proposed Model stands as a testament to the continual advancements in algorithmic development. The significance of precision, specificity, sensitivity, and accuracy in classification underscores these metrics’ critical role in assessing the reliability and effectiveness of machine learning models. Pursuing more accurate and robust classifiers as the field evolves remains a dynamic and ongoing endeavor.

## 5 Conclusion and future work

In conclusion, the Hybrid Models for Decalcify Cardiac CT (HMDC) introduced in this study have demonstrated exceptional efficacy in enhancing the clarity of cardiac CT images through robust decalcification. The achieved accuracy of 97.22% surpasses existing methods, showcasing the potential of this hybrid approach in significantly improving diagnostic precision in cardiovascular imaging. The success of HMDC lies in its innovative combination of deep learning techniques and traditional image processing methods, allowing for a synergistic enhancement of decalcification outcomes. The model’s ability to unveil clearer cardiac CT images holds promise for advancing diagnostic capabilities, particularly in scenarios where calcification poses challenges to accurate interpretation. While this study presents a ground-breaking solution, there are several avenues for future research and development. Further refinement of HMDC could involve optimizing hyperparameters, exploring additional data augmentation techniques, and investigating the model’s adaptability to diverse datasets. The scalability and generalizability of HMDC across different imaging modalities and patient demographics warrant careful consideration. Additionally, integrating interpretability tools and explainable AI methodologies can enhance the model’s transparency, providing insights into the decision-making process. Collaborations with medical practitioners and radiologists for real-world validation and clinical trials are essential to validating the practical utility and impact of HMDC in healthcare settings. The broader application of hybrid models in medical imaging, beyond decalcification, offers a rich area for exploration. Future research could explore the extension of HMDC principles to address challenges in other imaging domains, contributing to a more comprehensive toolkit for healthcare professionals. In conclusion, HMDC represents a significant advancement in cardiac CT imaging and lays the groundwork for continued innovation in hybrid models, fostering a transformative impact on diagnostic accuracy and patient care in cardiovascular health.

## Data Availability

Publicly available datasets were analyzed in this study. This data can be found here: https://www.kaggle.com/datasets/shivan118/healthcare-analytics.
